# Low-Volume Home Haemodialysis and In-Centre Haemodialysis: Comparison of Dialysis Adequacy in Obese Individuals

**DOI:** 10.7759/cureus.35054

**Published:** 2023-02-16

**Authors:** Adel A Alalwan, Aissar Abou Trabeh, Mohamed Mujahith SB Ahamed, Samuel Jones, Donald Adjorlolo, Robert Lewis, Nicholas Sangala

**Affiliations:** 1 Renal Medicine, Portsmouth NHS Hospitals, Portsmouth, GBR

**Keywords:** comorbid obesity, in-centre haemodialysis, home haemodialysis (hhd), nxstage system one, haemodialysis (hd)

## Abstract

Background

Although frequent low-flow, low-volume haemodialysis using the NxStage System One is now well-established as an option for home therapy of end-stage chronic kidney disease, its ability to deliver adequate dialysis in people with high BMI remains questionable. This doubt may lead to obese individuals being denied the potential benefits of this modality. To establish if this doubt is justified, we compared dialysis adequacy in two groups of obese individuals; one receiving standard thrice-weekly in-centre haemodialysis and the other receiving frequent haemodialysis at home using the NxStage System One.

Methods

This is a retrospective observational cohort study of 105 adult dialysis patients with obesity (BMI ≥ 30 kg/m2). All had been on dialysis for at least six months. Fifty-five patients receiving in-centre haemodialysis were compared with 50 patients receiving home haemodialysis using NxStage System One. Dialysis adequacy (standard Kt/V calculated by the Daugirdas equation) was compared between the two groups. The clinical characteristics, laboratory test results, and treatment regimens of each group were also compared.

Results

The in-centre haemodialysis group was older (63.6 ± 12.8 years vs. 58.5 ± 10.9 years, p=0.033) and had a higher Charlson comorbidity score (5.9 ± 2.1 vs. 4.5 ± 2.5, p=0.003). Standard Kt/V was significantly higher in the home haemodialysis group (2.4 ± 0.5) than in the in-centre haemodialysis group (2.2 ± 0.2) (p = 0.006). The mean serum inorganic phosphate was significantly lower in the home haemodialysis group than in the in-centre haemodialysis group (1.6 ± 0.4 mmol/l vs. 1.8 ± 0.5 mmol/l, p = 0.010). There were no statistically significant differences in the usage of antihypertensives, phosphate binders, or erythropoiesis-stimulating agents between the two groups.

Conclusions

In this study, dialysis adequacy (expressed as standard Kt/V) was superior to that of standard thrice-weekly in-center haemodialysis delivered by frequent low-volume home haemodialysis using the NxStage System One. Hesitancy about recommending frequent low-volume home haemodialysis to obese individuals is therefore unjustified.

## Introduction

Chronic Kidney Disease (CKD) is a major global public health problem affecting more than 700 million people and growing at 8% per year. The number of individuals with end-stage renal disease (ESRD) is also growing, particularly amongst those with obesity, diabetes, and hypertension [1-3]. Obese individuals (body mass index (BMI) ≥30 kg/m2) are less likely to be transplanted and are therefore often dependent on long-term dialysis. Achieving adequate dialysis in obese individuals is challenging, with many requiring longer and more frequent haemodialysis [3]. Home haemodialysis (HHD) offers a better quality of life, greater opportunity for rehabilitation and employment, and is more cost-effective than in-centre HD [4,5]. It is also associated with a lower risk of cardiovascular death and hospitalization, better control of hypertension, and reduced left ventricular mass [1,6]. HHD would be an ideal treatment for obese individuals with ESRD. Despite the advances in HHD, in-centre HD remains the most prevalent dialysis modality in the UK and around the world [2,7]. The NxStage System One dialysis machine is compact, portable, occupies less living space, and reduces the need for renovation in homes, meaning it is more accessible as a home treatment than traditional dialysis machines [8,9]. However, the effectiveness of low-flow, low-volume HHD delivered by NxStage System One in obese individuals is unclear, meaning many obese individuals are denied the opportunity to undergo HHD despite its numerous advantages [1,8].

Low-flow, low-volume HHD has additional sustainability advantages. This is particularly important in a time of financial restraint and concern about the environmental impact of health care. Traditional haemodialysis uses high dialysate flow rates (500 ml/min) and uses 120 litres of dialysate in a four-hour treatment, for a total of 360 litres a week for a typical thrice-weekly regimen. Hemodiafiltration uses even more water. In contrast, a typical NxStage System One prescription uses 25-30 litres of dialysate per treatment, six days per week, for a total of 150-180 litres of dialysate per week, significantly less than in-centre HD.

Given the apparent advantages of frequent low-flow, low-volume HHD using the NxStage System One over in-centre HD, it is important to establish if it delivers adequate treatment in obese individuals and whether concerns about its suitability in obese individuals are justified.

## Materials and methods

**Figure 1:**
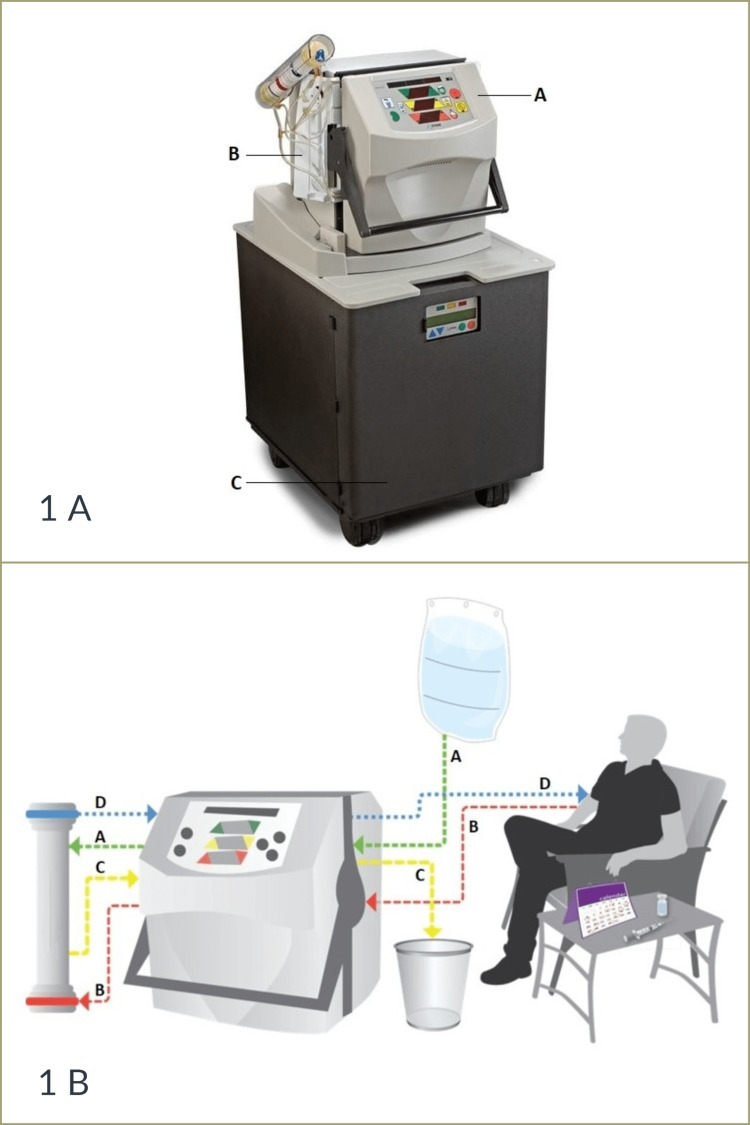
NxStage System One components and concept Figure 1A: NxStage System One components. (A) System One: the main unit that contains fluid pumps and system controls with an integrated, easy-to-use interface. (B) Cartridge: a single-use, gamma-sterilized component that usually houses an integrated dialyzer. (C) PureFlow SL: the dialysate preparation system that purifies tap water and then combines it with concentrate to create dialysate. Figure 1B: NxStage System One concept. (A) The dialysate solution (prepared using the PureFlow SL or in premixed bags) passes across the dialyzer (green arrow path). (B) the patient’s blood passes across the dialyzer in a counter-current direction (red arrow path). (C) Used dialysate, waste, and excess fluids removed from the blood are routed through a waste line into a sink, toilet, or drain (yellow arrow path). (D) The filtered blood returns to the patient’s circulation (blue arrow path).

A retrospective observational cohort study was conducted at the Wessex Kidney Centre in Queen Alexandra Hospital, Portsmouth, UK, over a period of four months (March to July 2022). The medical records of 105 dialysis patients with obesity (55 patients on in-centre HD and 50 patients on HHD) were reviewed. Adults over the age of 18 with a BMI≥30 kg/m2 who had been receiving thrice-weekly in-center HD or HHD for at least six months were eligible for the study. We excluded patients with an inadequate blood flow rate on dialysis (blood flow rate < 250 ml/min per session) and patients who had a possible active infection or inflammation (C-reactive protein value > 100 mg/L) at the time of data collection. All HHD patients utilised the low-flow, low-volume NxStage System One, which is illustrated in Figure 1 [10,11]. High-flux synthetic dialyzers were utilised in both modalities; the NxStage System One utilised a glycerine-free polyethersulfone membrane (PUREMA®H) [12], and the FX dialyzers with an advanced polysulfone membrane (Helixone®) were used in the in-centre HD group [13].

Clinical and laboratory data required for the study were gathered from the local electronic renal database. This included age, gender, height, weight, body mass index (BMI), primary renal diagnosis, co-morbidities, prior dialysis and renal transplantation history, and medication. HD-related factors included the number of sessions per week, duration of each session, blood flow rate, and vascular access. The latest records of pre-dialysis haemoglobin and serum biochemistry (sodium, potassium, adjusted calcium, phosphorus, C-reactive protein, and albumin) were obtained.

Dialysis adequacy was expressed using standard Kt/V_urea_ (std Kt/V), a hypothetical continuous clearance dependent on the urea nitrogen generation rate and the average pre-dialysis urea nitrogen in haemodialysis patients. The Daugirdas equation was used to calculate the standard Kt/V in this study. It uses a modelled urea volume and considers the ultrafiltration volume as well as the residual renal clearance. Adequate standard Kt/V is defined as a minimum target value of 2.12 per week [14]. This factor was calculated for all the study participants.

Statistical analysis

IBM Statistical Package for Social Sciences (SPSS) Version 26 (IBM Corp., Armonk, NY, USA) was used for data entry and analysis. Frequencies and percentages were computed for categorical variables, whereas means and standard deviations were computed for quantitative variables. The T-test was used to determine whether there is a significant difference in means between the two independent groups. The Chi-square test was used to determine whether there is a significant relationship between two categorical variables. Fisher's exact test was used in the two-by-two contingency table to test whether there is a significant relationship between two categorical variables in the case of small counts. In all statistical analyses, statistical significance was found when the p-value was less than 0.05.

## Results

The study included 55 patients on in-centre HD and 50 patients on HHD. The in-centre HD population was significantly older, had a higher Charlson comorbidity score (5.9 ± 2.1 vs 4.5 ± 2.5, p=0.003), and had a higher incidence of diabetes mellitus and cerebrovascular diseases. The two groups were both predominantly male (70.9% in in-centre HD and 78% in HHD). There were no statistical differences in body mass index (BMI), cause of primary renal disease, incidence of hypertension, or other co-morbidities between the two groups. The majority of HHD patients had previously received a different dialysis modality (in-centre HD in 86%; Table 1).

**Table 1:** Clinical characteristics of the study cohort by haemodialysis (HD) modality Note: All variables are presented as n (%) for categorical variables or as mean ± SD for continuous variables. In all statistical analyses, a p-value of less than 0.05 was considered statistically significant. TIA: transient ischaemic attack

Characteristics	Haemodialysis (HD) modality		p-value
	In-centre HD (n = 55)	Home HD (n = 50)	
Age (Mean ± SD)	63.6 ± 12.8	58.5 ± 10.9	0.033
Gender (n (%))			
Male	39 (70.9)	39 (78)	0.406
Female	16 (29.1)	11 (22)	
Weight (Kg) (Mean ± SD)	111.9 ± 15	112.9 ± 14.3	0.741
Height (cm) (Mean ± SD)	171.2 ± 9.4	176.1 ± 10.3	0.012
Body mass index (kg/m²) (Mean ± SD)	38.4 ± 5.9	36.8 ± 6.5	0.174
Primary renal diagnosis (n (%))			
Diabetes mellitus	26 (47.3)	15 (30)	0.074
Hypertensive nephrosclerosis	7 (12.7)	4 (8)	
Glomerular diseases	10 (18.2)	7 (14)	
Polycystic kidney disease	4 (7.3)	9 (18)	
Other inherited and congenital renal anomalies	0 (0)	4 (8)	
Obstructive uropathy	4 (7.3)	3 (6)	
Others / idiopathic	4 (7.3)	8 (16)	
Diabetes mellitus (n (%))	31 (56.4)	16 (32)	0.012
Hypertension (n (%))	47 (85.5)	39 (78)	0.322
Cardiovascular disease (n (%))	16 (29.1)	17 (34)	0.588
Peripheral vascular disease (n (%))	9 (16.4)	7 (14)	0.736
Cerebrovascular disease (stroke/TIA) (n (%))	6 (10.9)	0 (0)	0.028
Gout (n (%))	2 (3.6)	6 (12)	0.147
Malignancy (n (%))	7 (12.7)	8 (16)	0.632
Other co-morbidities (n (%))	14 (25.5)	13 (26)	0.949
Charlson comorbidity score (points) (Mean ± SD)	5.9 ± 2.1	4.5 ± 2.5	0.003
Duration of haemodialysis (Months) (Mean ± SD)	35.4 ± 24.1	33.1 ± 21.7	0.607
Prior dialysis modality (n (%))	0 (0)	45 (90)	<0.001
In-center haemodialysis (n (%))	0 (0)	43 (86)	<0.001
Home haemodialysis (n (%))	0 (0)	1 (2)	0.476
Peritoneal dialysis (n (%))	0 (0)	3 (6)	0.105
Prior renal transplantation (n (%))	5 (9.1)	10 (20)	0.111

As expected, the HHD group had a higher number of weekly HD sessions (5.6 ± 0.8) and shorter individual HD sessions (184.4 ± 43.8 minutes) (p < 0.001) (Table 2). Both groups had similar blood flow rates (351.8 ± 56.2 ml/min per session in the HHD group and 361 ± 53.4 ml/min per session in the in-centre HD group). 52.7% of in-centre HD patients had an arteriovenous fistula versus 64% in the HHD group. This difference was not statistically significant (Table 2).

**Table 2:** Haemodialysis-related factors in the study cohort by haemodialysis (HD) modality Note: All variables are presented as n (%) for categorical variables or as mean ± SD for continuous variables. In all statistical analyses, a p-value of less than 0.05 was considered statistically significant.

Haemodialysis-related factors	Haemodialysis (HD) modality		p-value
	In-centre HD (n = 55)	Home HD (n = 50)	
Number of haemodialysis sessions/week (Mean ± SD)	3 ± 0.1	5.6 ± 0.8	<0.001
Duration of haemodialysis session (minutes) (Mean ± SD)	238.2 ± 14	184.4 ± 43.8	<0.001
Blood flow rate per session (ml/min) (Mean ± SD)	361 ± 53.4	351.8 ± 56.2	0.392
Vascular access modality [n (%)]			
Tunnelled vascular catheter	25 (45.5)	17 (34)	0.488
Arteriovenous (AV) graft	1 (1.8)	1 (2)	
Arteriovenous (AV) fistula	29 (52.7)	32 (64)	

**Figure 2:**
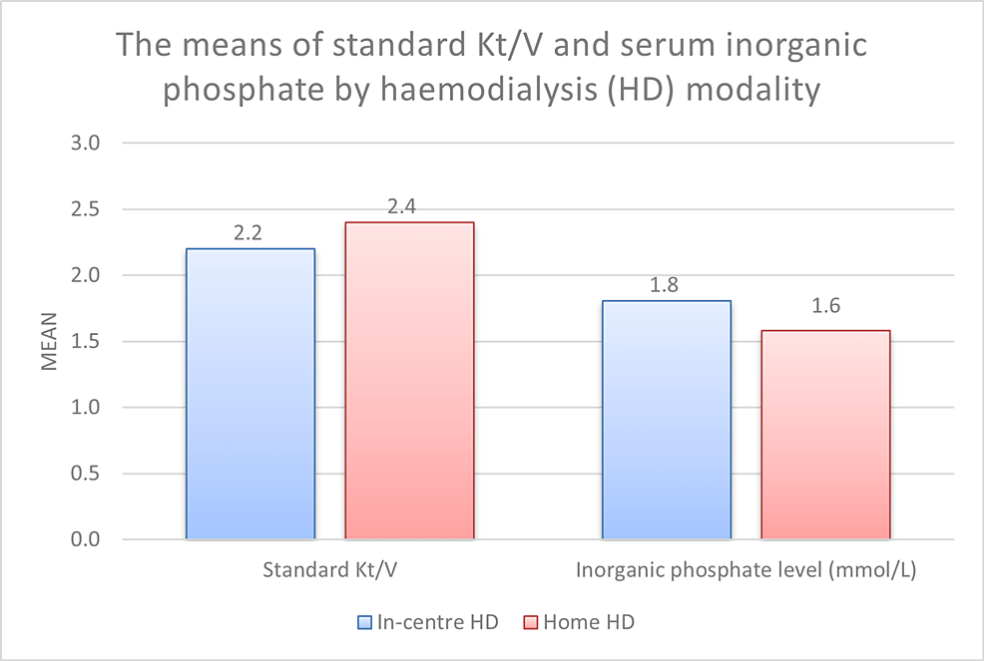
The means of standard Kt/V and serum inorganic phosphate by haemodialysis (HD) modality

The std Kt/V was significantly higher in the HHD group (2.4 ± 0.5) compared with the in-centre HD group (2.2 ± 0.2) (p = 0.006) (Table 3, Figure 2). In addition, the serum inorganic phosphate was significantly lower in the HHD group (1.6 ± 0.4 mmol/l) compared with the in-centre HD group (1.8 ± 0.5 mmols/l) (p = 0.010). There were no statistically significant differences in haemoglobin, serum potassium, and serum albumin levels between the two groups (Table 3).

**Table 3:** Dialysis adequacy (standard Kt/V) and laboratory parameters of the study cohort by haemodialysis (HD) modality Note: All variables are presented as mean ± SD for continuous variables. In all statistical analyses, a p-value of less than 0.05 was considered statistically significant.

Dialysis adequacy and laboratory parameters	Haemodialysis (HD) modality		p-value
	In-centre HD (n = 55)	Home HD (n = 50)	
Standard Kt/V (Mean ± SD)	2.2 ± 0.2	2.4 ± 0.5	0.006
Haemoglobin g/L (Mean ± SD)	108.4 ± 10.9	100.8 ± 28.5	0.082
Serum sodium mmol/L (Mean ± SD)	137.9 ± 3	137.2 ± 3.1	0.248
Serum potassium mmol/L (Mean ± SD)	4.8 ± 0.6	4.6 ± 0.6	0.258
Serum adjusted calcium mmol/L (Mean ± SD)	2.3 ± 0.2	2.3 ± 0.1	0.504
Serum inorganic phosphate mmol/L (Mean ± SD)	1.8 ± 0.5	1.6 ± 0.4	0.010
Serum c-reactive protein (CPR) mg/L (Mean ± SD)	10.1 ± 5	10 ± 5.4	0.930
Serum albumin g/L (Mean ± SD)	29.7 ± 7	30.8 ± 8	0.433

**Figure 3:**
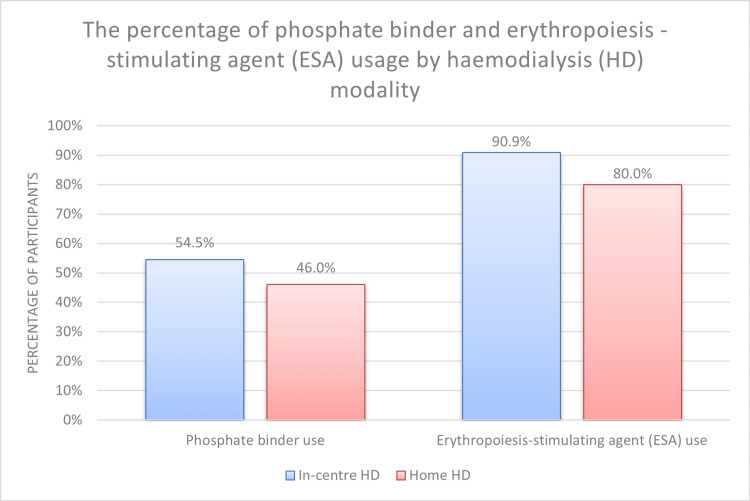
The percentage of phosphate binder and erythropoiesis-stimulating agent (ESA) usage by the haemodialysis (HD) modality

Furthermore, there were no significant differences in the number of antihypertensive medications used, the weekly dose of erythropoietin stimulating agent (ESA), and the usage of phosphate binders between the two groups. (Table 4, Figure 3).

**Table 4:** Medication use in the study cohort by haemodialysis (HD) modality Note: All variables are presented as n (%) for categorical variables or as mean ± SD for continuous variables. In all statistical analyses, a p-value of less than 0.05 was considered statistically significant.

Variables	Haemodialysis (HD) modality		p-value
	In-centre HD (n = 55)	Home HD (n = 50)	
Antihypertensive medication use [n (%)]	44 (80)	36 (72)	0.336
Number of antihypertensive medication/day (Mean ± SD)	2.1 ± 1.2	2 ± 0.8	0.682
Phosphate binder use [n (%)]	30 (54.5)	23 (46)	0.382
Erythropoiesis-stimulating agent (ESA) use [n (%)]	50 (90.9)	40 (80)	0.111
ESA units per week (Mean ± SD)	12640 ± 11573.7	11662.5 ± 10916.6	0.684

## Discussion

Haemodialysis patients with obesity (BMI ≥30 kg/m2) are often deemed unsuitable for transplantation due to the increased risk of post-transplant complications and poor transplant outcomes [15-6], and as a result, may remain on dialysis for many years. HHD is cost-effective and facilitates adaptation of the dialysis prescription for enhanced hemodynamic stability and improved overall quality of life [16,17]. HHD using conventional three times-a-week sessions usually requires alterations to the patient’s home to accommodate the dialysis machine and a reverse osmosis system. In contrast, NxStage System One is compact and portable, occupies less living space, and reduces the need for home alteration. However, it employs a low volume of lactate-buffered ultrapure dialysate per session and inverts the traditional ratio between dialysate and blood flow rates [1,8,9]. The ability of this system to deliver adequate dialysis to obese individuals has been questioned, and this presents a barrier to obese individuals benefiting from HHD. This is the first study to directly compare dialysis adequacy in obese patients using the NxStage System One and traditional thrice-weekly HD. Our findings support the use of NxStage System One in obese patients by establishing that it can deliver greater dialysis adequacy (as measured by std Kt/V) compared with in-centre HD (Figure 2). Our findings are consistent with the Knowledge to Improve Home Dialysis Network in Europe (KIHDNEy) cohort which recruited 129 haemodialysis patients using NxStage System One. It concluded that intensive HHD with low-flow dialysate delivers adequate urea clearance and good biochemical outcomes in patients across three body mass categories (normal, overweight, and obese) [1].

Frequent, low-volume HHD appears to be a viable and effective treatment modality in obese patients; however, there are still challenges to overcome in this population. Obese individuals who are older in age and/or diabetics tend to have a higher rate of complications and decreased abilities and skills. They often require additional help at home and struggle with the technical aspects of HHD, such as performing self-punctures of their fistulas [16,18].

In our study, serum inorganic phosphate levels were significantly lower with frequent low-volume HHD using the NxStage System One compared with conventional in-centre HD. This finding is consistent with a study by Brunati et al., which showed that NxStage System One achieved higher weekly phosphate removal than traditional in-centre HD (3,488 ± 1,181 mg vs. 2,634 ± 878 mg, p < 0.003) [19]. In addition, Zaritsky et al. revealed that fibroblast growth factor 23 (FGF23) levels were lower in HHD patients undergoing short daily haemodialysis using the NxStage System One, in contrast to in-centre HD (823 RU/mL vs. 2521 RU/mL, p < 0.01) [20]. FGF23 levels can estimate the cumulative phosphate burden more sensitively compared to single or even multiple serum phosphorus determinations [20]. Although the use of phosphate binders appeared lower in the HHD patients than in the in-centre HD group in our study, this difference did not reach statistical significance (Figure 3).

In terms of anaemia management, although usage of erythropoietin-stimulating agents (ESA) was relatively lower in HHD patients compared to in-centre HD (Figure 3), we did not report any statistically significant differences between both modalities in haemoglobin levels, ESA usage, or weekly units of ESA. The effects of daily haemodialysis on erythropoiesis remain debatable with variations in haemoglobin or haematocrit levels as well as ESA utilization [1,21,22]. These parameters can be difficult to interpret due to the increased blood loss through the dialysis circuit and the resulting iron deficiency in HHD patients [22].

In our study, we found that the number of patients on antihypertensives and the number of antihypertensive medications per day were both lower in the HHD group; however, this did not reach statistical significance (Table 4). In the KIHDNEy cohort, there was a significant decline in antihypertensive medication usage between baseline and follow-up of patients on low-volume HHD [1]. These findings can be partly explained by frequent, regular haemodialysis and better solute clearance in HHD, which can lead to lower interdialytic weight gain and a reduction in pre-dialysis blood pressure [23].

Limitations

Our study has a number of limitations, which we acknowledge. One is the lack of residual urine output data, which is important in the accurate calculation of std Kt/V. Notwithstanding this, residual renal function will not change the adequacy delivered by dialysis alone and would therefore not greatly affect the comparison. We also recognise the limitations of std Kt/V as a measure of adequacy. Although this is widely accepted as the standard measure [24], a full assessment of dialysis adequacy should include other factors that have not been fully explored in our study.

## Conclusions

In this study, frequent low-flow, low-volume HHD using NxStage System One had superior dialysis adequacy (assessed using standard Kt/V) compared to that attained by standard thrice-weekly in-centre HD. It also had a significant impact on lowering serum inorganic phosphate levels in these patients. The concern that the dialysis adequacy delivered by frequent low-flow, low-volume HHD is insufficient for obese individuals is therefore unfounded, and they should not be denied the advantages that this modality may bring. In addition, awareness of this dialysis modality is crucial among nephrologists and nephrology trainees to allow for early referrals and faster enrollment of these patients in training programs.
